# Membrane Fusion and SNAREs: Interaction with Ras Proteins

**DOI:** 10.3390/ijms23158067

**Published:** 2022-07-22

**Authors:** Azzurra Margiotta

**Affiliations:** Independent Researcher, 0364 Oslo, Norway; azzurramarg@libero.it

**Keywords:** Ras proteins, Rab proteins, SNAREs, interaction, membrane fusion, vesicle trafficking

## Abstract

The superfamily of Ras proteins comprises different molecules belonging to the GTPase family. They normally cycle between an active state bound to GTP which activates effectors while the protein is membrane-associated, and an inactive GDP-bound state. They regulate the intracellular trafficking and other cellular processes. The family of Rab proteins includes several members and they have been found, among other Ras proteins, to be fundamental for important biological processes, such as endocytosis and exocytosis. SNARE proteins control the fusion of vesicles by forming quaternary complexes which are divided into two small groups on the two different compartments. Generally, the association of three SNARE proteins on the donor compartment with the one on the target compartment determines the formation of the SNARE complex, the opening of the fusion pore and the formation of one single bigger vesicle. Interestingly, novel interactions between other molecules involved in intracellular trafficking, endosomal fusion and maturation have recently been found, such as the interaction between invariant chain and the Qb SNARE vti1b, and more functional connections between Rab proteins and SNAREs are supposed to be fundamental for the regulation of membrane fusion.

## 1. Introduction

Proteins that belong to the superfamily of small guanosine triphosphates, named small GTPases, G-proteins, or the Ras superfamily, take part in most of the biological processes occurring in cell biology. These proteins switch from an active GTP-bound form to an inactive GDP-bound form after the hydrolysis of GTP to GDP. The superfamily comprises five families that are well conserved in eukaryotes, which are named Ras, Rho, Rab, Arf/Sar, and Ran [1].

Ras-associated binding (Rab) proteins are guanine nucleotide-binding proteins which are regulated by several key factors, including GTPase-activating proteins (GAPs) and GTP-exchange factor proteins (GEFs) for GTP hydrolysis and GDP dissociation, respectively [2]. More than 60 human Rab proteins are known so far, and their function is related to the interaction with different proteins named effectors that can modulate different processes. Rab and Rab-associated proteins are localized in several parts of the cell and they regulate different steps of intracellular trafficking [3].

Ras proteins are activated by extracellular stimuli and, in turn, they regulate intracellular signaling. Their main function is to control gene transcription, cell growth and differentiation. Moreover, the human oncogenic members regulate cell proliferation, differentiation, morphology, and apoptosis [4]. The Rho family is responsible for regulating the cytoskeleton, cell polarity, cell cycle progression, hematopoiesis and gene expression [5], while the Arf family is involved in regulating vesicle trafficking [4]. Finally, Ran proteins are involved in regulating the nuclear transport [6].

SNARE proteins are mainly membrane proteins that work in a complex and that are required for regulating docking of granules and vesicles to target membranes, including the plasma membrane, and membrane fusion which is fundamental for many biological processes, such as viral infection, cell fertilization, intracellular transport and neurotransmitter release. The fusion machinery is based on the participation of several molecules; however, the SNARE proteins are grouped into *v*-SNAREs (vesicle-SNARE) and *t*-SNAREs (target-SNARE) which are located on the two different membranes and that assemble in one unique complex when the fusion process is complete [7]. For membrane fusion, three or mainly four SNAREs form the complex, giving rise to a ternary or quaternary complex, respectively. They are located at different compartments and they mediate diverse biological events. Moreover, they are grouped in R-, Qa-, Qb-, Qb-c-, or Qc-SNAREs depending on the residue that it is exposed in the formation of the zero ionic layer in the assembled core SNARE complex. Generally, each complex is made of a Qa-, a Qb-, a Qc- and a R-SNARE. For ternary complex, a Qb-c-SNARE replaces the Qb- and Qc-SNAREs [7].

**Figure 1:**
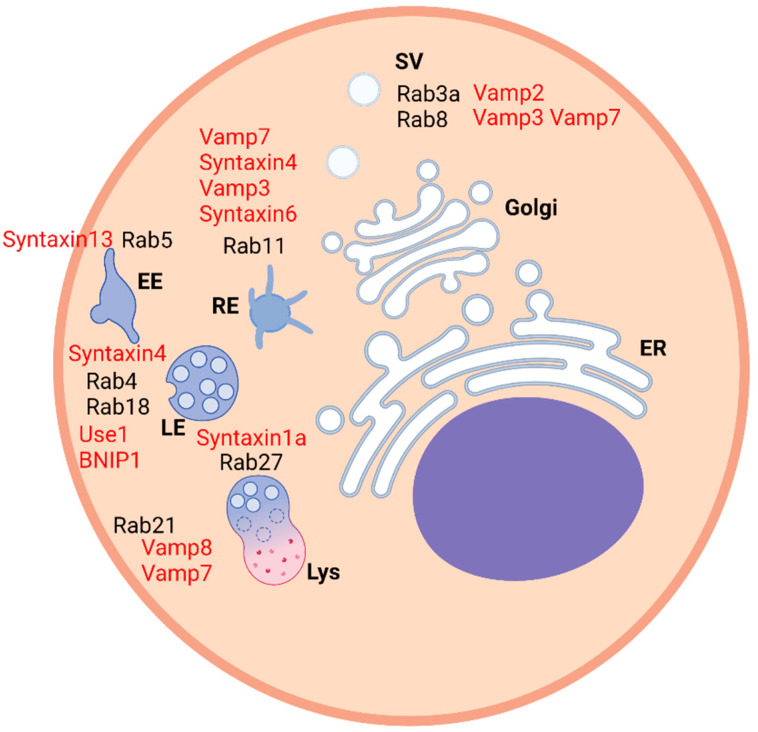
Scheme representing a generic animal cell. Rab proteins are located in the right compartments and the relative interacting SNARE proteins are labeled in red. EE—early endosome, RE—recycling endosome, LE—late endosome, Lys—lysosome, ER—endoplasmic reticulum, SV—secretory vesicle.

Interestingly, several molecules are involved in the regulation of intracellular trafficking and the interaction between a SNARE molecule, named Vti1b, and a transmembrane protein, named invariant chain (Ii), has been detected recently to be a fundamental association that could explain how the role of Ii in the endosomal fusion and maturation was exerted [8]. Similarly, some interactions between Ras proteins and SNAREs demonstrate how they can work together and how they regulate membrane fusion and intracellular trafficking (Table 1) (Figure 1).

## 2. Interaction between Rab and SNARE Proteins

The interaction between proteins can be either direct or indirect, meaning that it is mediated by other molecules. Several biochemical techniques, such as co-immunoprecipitation, pull-down, yeast-two-hybrid, and others, can be used to identify these associations. For many years, the two classes of proteins, Ras and SNAREs, have been related to different aspects of intracellular trafficking. Rab proteins are normally involved in recruiting effectors, they are associated with cytoskeletal elements and they mediate the first specific tethering event between a vesicle and its target compartment [27]. Meanwhile, SNARE proteins mediate fusion by bridging the opposite membranes before fusion, regulating the formation of the full complex, modulating its assembly and disassembly on the membrane and the opening of the fusion pore [28].

### 2.1. Yeast-Two-Hybrid as a Technique to Detect Interesting Interaction Partners

In the paper by Grote and Novick, a novel interaction was discovered in yeasts between ypt1p, a member of the Ypt/Rab family of small GTP-binding proteins, and sec5p, the exocyst complex component 2, which is required before SNARE complex assembly, in the cis-Golgi compartment. Moreover, some other mutants of Rab homologs interact with yeast snare proteins. The ypt1p mutant was able to interact with pep12 (*t*-snare), snc (*v*-snare) and sso (*t*-snare) while the ypt32p mutant interacted with pep12, snc and sso. Furthermore, the mutant of sec4p, a small monomeric Ras-related GTP-binding protein that regulates secretory vesicles and transport from the Golgi apparatus to the cell surface, interacted with sso, whereas the ypt7 mutant interacted with pep12, snc and sso [2]. These associations need to be confirmed with other techniques that could prove the real interaction and functional link between the proteins; however, the yeast-two-hybrid assay showed interesting candidates that could be of interest for further studies and also for testing their homologs in mammalian cells. Interestingly, another interaction proven by two-hybrid yeast analysis is that demonstrated by Ungermann and colleagues between vam7p (the vacuolar SNAP-23/25 homolog) and ypt7p (the vacuolar Rab/Ypt). Their interaction is necessary in order to initiate the docking process and vam7p seemed to represent an important molecule that regulates ypt7p. Therefore, vam7p is an important link for both the priming step, where it is released from the SNARE complex, and docking. At the same time, ypt7p is required to keep vam7p on the vacuole [26].

An interesting study demonstrated that a prenylated rab acceptor (pra1) was able to regulate membrane fusion mediated by rab3a or rab1 and vamp2. Deletion analysis on PRA1 indicated that the critical rab- and vamp2-interacting residues reside in two regions: the amino-terminal residues 30–54 and the extreme carboxyl-terminal domain. This dual rab and vamp2 binding characteristic suggests that PRA1 may serve to link these two proteins in the control of vesicle docking and fusion. Rab3a and vamp2 do not bind together to pra1; therefore, they do not form a ternary complex. However, rab3a could displace vamp2 in the complex together with pra1 by determining an alteration in the conformation of pra1 and therefore decreasing affinity for vamp2. Pra1 is present both in the cytosol and in the membrane compartment and it is also able to inhibit the extraction of membrane-bound rab3a by GDI. It favors membrane retention of rab while GDI maintains rab soluble in the cytosol therefore they have an opposite effect and they determine the amount of active rab protein [29]. Therefore, the role of pra1 may be relevant for the regulation of specific steps of intracellular trafficking. Interestingly, pra1 also interacts with rab5 and rab6 [30].

### 2.2. Interaction between SNAREs and Rab Proteins in Intracellular Trafficking

Syntaxin 13 localizes on early and tubular recycling endosomes and directly interacts with EEA1. The rab 5-dependent coordination between EEA1 and syntaxin 13 could be fundamental for docking and subsequent assembly and activation of the fusion machinery, therefore making EEA1 a molecular link between the rab machinery regulating endosome tethering and SNARE-mediated fusion. When the interaction between EEA1 and syntaxin 13 is abolished, the endosome fusion is inhibited. However, when rab5 is active and the effector proteins are recruited, they oligomerize with components of the SNARE machinery and the transition from membrane docking to fusion occurs [16].

A process that is based on membrane fusion is autophagy, which consists of the transport of material to autophagosomes where they are responsible for its degradation. Active rab7 is located on late endosomes and lysosomes and mediates the fusion of these with autophagosomes by interacting directly with its effector EPG5, which is a fundamental molecule as it interacts with SNARE proteins and facilitates the formation of the complex that determines the fusion of vesicles. In particular, EPG5 binds both vamp7/8 which are localized on late endosomes/lysosomes and syntaxin 17, which is located on autophagosome. Interestingly, syntaxin 17, which is a transmembrane protein, is assembled to snap 29, which does not contain this domain and it is a cytosolic protein. After the activation of rab7, the binding of EPG5 and the recruitment of these SNAREs and their assembly, the fusion between lysosomes and autophagosomes occurs, giving rise to autolysosome. Moreover, EPG5 interacts directly also with LC3/LGG-1 on autophagosomes. Given the importance of this molecule in this process, when EPG5 does not work properly, non-specific fusion of autophagosomes with other types of vesicles occurs and non-degradative enlarged vesicles are formed. In case of mutation in the EPG5 gene, the multisystem disorder Vici syndrome might develop [10].

Rab21 is localized at several compartments, such as recycling endosomes, plasma membrane, endocytic compartments and lysosomes. It interacts with vamp7 and vamp8 in endosome–lysosome fusion and in autophagosome–lysosome fusion. The role of rab21 in autophagy has been established thanks to the use of specific markers such as p62 and Atg8 and, in particular, rab21 functionally interacts with vamp7 in this process while the interaction with vamp8 was variable and related to starvation when rab21 activity was enhanced. In relation to the role of rab21 in other steps of intracellular trafficking, its major role is during the homotypic fusion of late endosomes together with syntaxin 7, syntaxin 8, vti1b and vamp8 [22].

Rab32 is involved in the trafficking from vesicles to lysosome-related organelles (LROs), such as melanosomes, autophagosomes and platelet dense granules. Rab32 is important in the regulation of the transport from endosomes to plasma membrane. An effector of rab32/38 is a nucleotide exchange factor GEF named varp (vps9 domain and ankyrin-repeat-containing protein), which also interacts with vamp7. Its recruitment to endosomes is dependent on its interaction with vps29, which has been proven by co-immunoprecipitation. Interestingly, varp binds in vitro at the same time to vps29, rab32/38 and vamp7. This might be relevant for the regulation of several biological events. In particular, the pathway for transporting GLUT1 includes rab32, varp and vamp7 [25].

### 2.3. SNAREs and Rab Proteins in Ciliogenesis

Rab8 is involved in ciliogenesis and colocalizes with IFT20, a component of the intraflagellar transport (IFT) system, in rab11-positive endosomes of non-ciliated T-cells. The delivery of recycling T-cell receptors (TCRs) to the immune synapse is regulated by the GTPase, which recruits vamp3, and their interaction has been confirmed by co-immunoprecipitation. Rab8 is located at the base of the cilium in NIH-3T3 cells where it also regulates ciliary growth. Vamp3 and the relative SNARE complex facilitate the transport of the endosomes carrying the protein smoothened (Smo) cargo to the immune synapse and the primary cilium [17].

Interestingly, the small GTPase arf4 binds the VxPx ciliary-targeting signal (CTS) of the light-sensing receptor rhodopsin in primary cilia of the retinal rod photoreceptor cells. An important process in order to avoid the photo-oxidative damages is the transport of the cargoes in ciliary trafficking. In the pathway regulated by this GTPase, the rab11a-FIP3-rabin8 complex controls the assembly of the rab11a-rabin8-rab8 ciliary-targeting module and Rab proteins interact with vamp7. This SNARE protein forms a complex with syntaxin 3 and SNAP-25 in order to allow ciliogenesis. The interaction between vamp7 and the Rab proteins is dependent on their binding to a specific domain of the SNARE. If vamp7 binds to the rab11-rabin8 complex, the SNARE protein is in the inactive closed conformation as the SNARE domain folds over the interaction domain and a conformational change occurs. A phosphorylation event determines the removal of the potential steric hindrance, enabling the rab11-rabin8-rab8 module to interact with vamp7 [18].

### 2.4. Interactions between Rab Proteins and SNARE Proteins during Cell Migration

**Figure 2:**
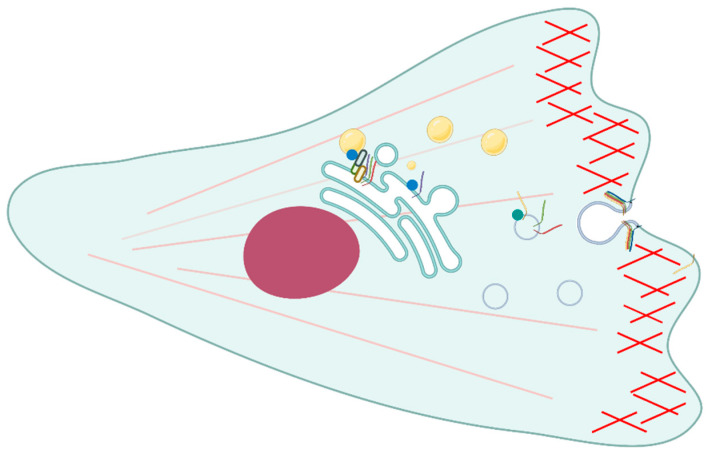
Scheme representing the interactions between Rab proteins and SNAREs during cell migration in mammalian cells. At the plasma membrane, Rab11 (green spot)-positive vesicles containing syntaxin 4 (yellow bar) fuse after SNARE complex formation. This transport is in association with the regulation of actin cytoskeleton and cortical actin (short red filaments). Interestingly, at the ER (green compartment), Rab18 interacts with syntaxin 18 (violet bar) through the mediation of a protein complex in order to regulate the formation of lipid drops (yellow circles).

Several interactions between Rab proteins and SNAREs have been detected during cell migration (Figure 2). Syntaxin 4 is a *t*-SNARE linked with cytoskeletal elements and it has been detected at the plasma membrane area in relation to the cortical actin cytoskeleton. In fact, it is important for secretion at the actin-rich areas of plasma membrane and may be recycled through intracellular vesicles. Syntaxin 4 colocalized together with rab11 on recycling vesicles and on the periphery of the cell. This colocalization persists also when N-ethylmaleimide (NEM), a drug that is able to dissociate the SNARE complex, is used, therefore the two proteins are supposed to interact. Moreover, the association is unchanged even in the case of different structures of actin filaments. In fact, the association is present when the actin is both in the form of actin bundles and stress fibers and it is important for the regulation of membrane trafficking at the plasma membrane. However, a direct interaction between rab11 and syntaxin 4 has to be proven with biochemical methods [19].

Regarding cell migration and adhesion, rab18 is able to interact with the endoplasmic reticulum (ER) and intracellular vesicles and it mediates trafficking to the plasma membrane [31]. Moreover, it has been found that rab18 is fundamental for the interaction with several other molecules involved in the endoplasmic reticulum and lipid droplets contact, such as syntaxin 18. The aim of this interaction is to regulate the lipid droplets growth and maturation and occurs through the action of the ER-associated NAG-RINT1-ZW10 (NRZ) tethering complex. Other ER-localized SNARE proteins associated with this process are Use1 and BNIP1. During the process of lipid droplet formation, neutral lipids are acquired from the ER by associating with it several times and the three SNAREs are recruited on the newly formed lipid droplets [21].

Interestingly, it has been found that another Rab protein named rab21 interacts with TI-VAMP/VAMP7 through varp [23]. This finding was related to yeast two-hybrid screenings using several libraries that confirmed varp as a new interactor of TI-VAMP/VAMP7. This molecule is a v-SNARE involved in PC12 neurite growth and is present in neurons in primary culture where it regulates the exocytic pathway of molecules such as β1 integrins, and cell adhesion, cell migration and phagocytosis. The three proteins co-localized in the perinuclear region and partially along the neurites in differentiated PC12 cells and in mouse hippocampal neurons. Nevertheless, in the peripheral region of the growth cone, varp and rab21 were mainly absent while TI-VAMP was prevalently located in filopodia. Varp is fundamental for the functional role of rab21 and TI-VAMP. The inactive form of rab21 was normally concentrated in the TGN while TI-VAMP regulated the exocytosis of post-Golgi vesicles. Varp is necessary for establishing a functional link between the two proteins most likely at TGN. Moreover, it seems that the activation of rab21 mediates the extension of long neurites [23].

Interestingly, there are cholesterol-sensitive SNAREs that play a role in cell migration and invasion. In Chinese hamster ovary (CHO) Niemann-Pick type C1 (NPC1) mutant cell lines and human NPC1 mutant fibroblasts, it has been proven that altered levels of cholesterol at the TGN/endosome boundaries determine syntaxin 6 accumulation into vamp3, transferrin, and Rab11-positive recycling endosomes (REs). Therefore, the SNARE complex is formed and the fusion of the compartments inhibits the recycling of αVβ3 and α5β1 integrins and cell motility and adhesion. Other molecules and events are affected, such as focal adhesion kinase (FAK), focal adhesion sites and directional migration toward fibronectin (FN) [32].

### 2.5. Membrane Fusion in Exocytosis

It is important to report that a molecule named granuphilin, which is supposed to work as a regulator in the exocytotic pathway, directly connects two fundamental vesicle transport proteins, Rab proteins and SNAREs. In particular, Torii and colleagues found that rab27a indirectly binds syntaxin 1a through granuphilin, a protein expressed mainly in pancreatic beta cells and pituitary tissue, at the N-terminal. The interaction between rab27a and granuphilin has been proven by several techniques, such as yeast two-hybrid, in vitro binding and coimmunoprecipitation experiments. They both co-localize on the membrane of insulin granules. Granuphilin directly binds syntaxin 1a on the plasma membrane and it is regulated by rab27a. Syntaxin 1a is in the closed conformation during this interaction; therefore, the SNARE complex cannot be formed. The importance of granuphilin is related to insulin secretion in MIN6 mouse insulinoma cell lines. Indeed, its basal secretion is increased when granuphilin is significantly overexpressed and inhibited when syntaxin 1a interacts with granuphilin in the closed conformation or when it is induced by high levels of K^+^ [24].

### 2.6. Neurotransmitter Receptor Recycling

GRASP-1 is a neuron-specific molecule fundamental in the event of neurotransmitter receptor recycling. Moreover, it functions also in the regulation of the normal morphology of dendritic spines and for synaptic plasticity. GRASP-1 interacts through its N-terminal domain with rab4 and to syntaxin 13 with its C-terminal domain. The role of GRASP-1 is to segregate rab4 from rab5-positive early endosomes and to coordinate the coupling to rab11-positive recycling endosomes via the interaction with syntaxin 13. Interestingly, rab4, rab11 and GRASP-1 colocalize on large endosomal structures where rab11 is also located at dynamic tubules. Caspase-3 cleavage can separate rab4 from syntaxin 13 and disrupt the interaction between rab4 and rab11 endosomes [15].

## 3. Interaction between Other RAS and SNARE Proteins

Leucine-rich repeat kinase 2 (LRRK2) belongs to the ROCO superfamily of proteins, characterized by a Ras-of-complex (Roc) GTPase domain and a C-terminal-of-Roc (COR) domain. This protein is involved in cilium formation. Moreover, LRRK2 is also involved in exocytosis of synaptic vesicles by interacting indirectly with SNAP-25 (synaptosomal-associated protein-25), which is a positive regulator of neurite length. This association is mediated by snapin, a SNAP-25 interacting protein, which is phosphorylated by LRRK2. When LRRK2 phosphorylates snapin, this molecule, in turn, interacts less with SNAP-25. This leads to decreased interaction of synaptotagmin-1 with the complex SNAP-25–syntaxin 1 and vamp2 and altered neurotransmitter release. Interestingly, the interaction of LRRK2 with rab5b slowed endocytosis of synaptic vesicles. From a clinical point of view, mutated LRRK2, corresponding to the PARK8 gene, has been linked to the familial Parkinson’s disease (PD) in an autosomal-dominant manner. Interestingly, both overexpression of the wild-type (WT) and PD-specific mutants of LRRK2 caused increased protein aggregation, decreased neurite length and branch number and increased oxidative stress-induced neurotoxicity [13].

Another interesting link is between the GTPase Arf and the SNARE protein snc1,2. In fact, this v-SNARE mediated endocytosis and exocytosis and interacted physically and genetically with Gcs1, an Arf GAP, which is important to mediate Golgi-ER and post-Golgi vesicle transport in yeast. The interaction between the SNARE protein and the GAP determined the binding of Arf and the recruitment of the coatomer. In particular, Gcs1 facilitated the recruitment of snc1,2 in COPI recycling vesicles and subsequent endosome-Golgi sorting [15]. Other Arf GAPs, Glo3p and Gcs1p, are responsible for binding to ER-Golgi v-SNAREs and, then, recruiting Arf1p and coatomer, that together form the COPI coat. Coat proteins may be necessary for the uptake of v-SNAREs into vesicles. For example, the v-SNAREs Bet1p and Bos1p bind specifically to components of the COPII coat [12].

In pancreatic beta cells, a direct interaction has been detected between both forms of cdc42, active and inactive, and the N-terminal domain of vamp2, which regulates SNARE-dependent insulin granule exocytosis. This association is linked to cortical F-actin reorganization and it is based on a mechanism involving the activation of the GTPase by glucose and the subsequent formation of the SNARE complex. Interestingly, syntaxin 1A and SNAP25 bind vamp2 at the plasma membrane. The disruption of the interaction between the SNARE complex and F-actin at the plasma membrane is necessary for the glucose-stimulated insulin secretion [9].

## 4. Conclusions

Several molecules are involved in the regulation of intracellular trafficking and many classes of proteins have been associated with different functions in this process.

First of all, tethering proteins are molecules able to bridge a vesicle to a target membrane and to regulate the fusion of vesicles [33]. Both Rab proteins and phosphoinositides are fundamental for membrane remodeling and regulation of membrane trafficking [34]. Phosphoinositedes have also a role in this function in relation to SNAREs as PI(3)P binds the SNARE protein vam7p and facilitate membrane fusion. Moreover, phosphoinositedes promote SNARE complex remodeling [35].

However, not many interactions and associated functional roles between SNAREs and Rab proteins have been identified in the past. Interestingly, in recent years more direct interactions and indirect associations between the two classes of proteins have been reported and presented in this manuscript. These interactions have been proven both in yeasts and mammalian cells, proving that the association is possible in different cell systems. Moreover, both in vitro and in vivo studies have been performed and coupling docking and fusion events is the major finding of these experiments. In several cases, the association between Rab proteins and SNAREs was mediated by a novel molecule which was working as a tethering factor, while in other cases, the link between the two classes of proteins was not known. Therefore, more studies in the future will be useful in order to define the model underlying this event.

## Figures and Tables

**Table 1:** Ras and SNARE proteins interact together. Findings regarding the proven interactions between the two classes of proteins are reported.

Ras Protein	SNARE	Reference
Cdc42	Vamp2	[9]
EPG5	Vamp7/8	[10]
Gcs1	Snc1,2	[11]
Glo3p and Gcs1p	*v*-SNAREs	[12]
LRRK2	SNAP-25	[13]
Rab3a	Vamp2	[14]
Rab4	Syntaxin4	[15]
Rab5	Syntaxin13	[16]
Rab8	Vamp3	[17]
	Vamp7	[18]
Rab11	Vamp7	[18]
	Syntaxin4	[19]
	Vamp3	[20]
	Syntaxin6	[20]
Rab18	Use1 BNIP1	[21]
Rab21	Vamp8	[22]
	Vamp7	[23]
Rab27a	Syntaxin 1a	[24]
Rab32/38	Vamp7	[25]
Ypt1p mutant	Pep12 Snc Sso	[2]
Ypt32p mutant	Pep12 Snc Sso	[2]
Sec4p mutant	Sso	[2]
Ypt7 mutant	Pep12 Snc Sso	[2]
Ypt7p	Vam7p	[26]
